# Inhibition of Mitochondrial Division Attenuates Cisplatin-Induced Toxicity in the Neuromast Hair Cells

**DOI:** 10.3389/fncel.2017.00393

**Published:** 2017-12-12

**Authors:** Jonathon W. Vargo, Steven N. Walker, Suhasini R. Gopal, Aditi R. Deshmukh, Brian M. McDermott Jr., Kumar N. Alagramam, Ruben Stepanyan

**Affiliations:** ^1^Department of Otolaryngology—Head and Neck Surgery, University Hospitals Cleveland Medical Center, Cleveland, OH, United States; ^2^Department of Biology, Case Western Reserve University, Cleveland, OH, United States; ^3^Department of Neurosciences, Case Western Reserve University, Cleveland, OH, United States; ^4^Department of Genetics and Genome Sciences, Case Western Reserve University, Cleveland, OH, United States

**Keywords:** cisplatin, mdivi-1, hair cells, zebrafish, mechanotransduction, mitochondria

## Abstract

Cisplatin and other related platinum antineoplastic drugs are commonly used in the treatment of a variety of cancers in both adults and children but are often associated with severe side effects, including hearing loss. Cisplatin’s ototoxic effects are multifaceted, culminating in irreversible damage to the mechanosensory hair cells in the inner ear. Platinum drugs act on cancerous cells by forming nuclear DNA adducts, which may initiate signaling leading to cell cycle arrest or apoptosis. Moreover, it was reported that cisplatin may induce mitochondrial DNA damage in non-cancerous cells. Therefore, protecting mitochondria may alleviate cisplatin-induced insult to non-proliferating cells. Thus, it is important to identify agents that shield the mitochondria from cisplatin-induced insult without compromising the anti-tumor actions of the platinum-based drugs. In this study we tested the protective properties of mitochondrial division inhibitor, mdivi-1, a derivative of quinazolinone and a regulator of mitochondrial fission. Interestingly, it has been reported that mdivi-1 increases the apoptosis of cells that are resistant to cisplatin. The ability of mdivi-1 to protect hair cells against cisplatin-induced toxicity was evaluated in a fish model. Wild-type (Tübingen strain), *cdh23* mutant, and transgenic *pvalb3b*::GFP zebrafish stably expressing GFP in the hair cells were used in this study. Larvae at 5–6 days post fertilization were placed in varying concentrations of cisplatin (50–200 μM) and/or mdivi-1 (1–10 μM) for 16 h. To evaluate hair cell’s viability the number of hair bundles per neuromast were counted. To assess hair cell function, we used the FM1-43 uptake assay and recordings of neuromast microphonic potentials. The results showed that mdivi-1 protected hair cells of lateral line neuromasts when they were challenged by 50 μM of cisplatin: viability of hair cells increased almost twice from 19% ± 1.8% to 36% ± 2.0% (*p* < 0.001). No protection was observed when higher concentrations of cisplatin were used. In addition, our data were in accord with previously reported results that functional mechanotransduction strongly potentiates cisplatin-induced hair cell toxicity. Together, our results suggest that mitochondrial protection may prevent cisplatin-induced damage to hair cells.

## Introduction

Cisplatin and other related platinum drugs are common antineoplastic agents that are used in the treatment of a variety of cancers in both adults and children (for a review see Jamieson and Lippard, 1999). However, these drugs are associated with various side effects including nephrotoxicity and ototoxicity (for review see Rybak et al., 2009; Schacht et al., 2012; Karasawa and Steyger, 2015; Francis and Cunningham, 2017). Although nephrotoxicity can be managed to some extent (Cornelison and Reed, 1993; Wong and Giandomenico, 1999), mitigating ototoxicity in patients treated with cisplatin remains an unmet medical need (Brock et al., 2012; Schacht et al., 2012; Karasawa and Steyger, 2015). The platinum drugs act on cancerous cells mainly by forming adducts within the DNA (Huang et al., 1995; Jamieson and Lippard, 1999) and, possibly, by increasing reactive oxygen species (ROS) levels (Kopke et al., 1997; Rybak et al., 1999; Devarajan et al., 2002). In addition, cisplatin leads to cytotoxicity in normal cells that are not actively proliferating, inducing mitochondrial DNA damage and ROS elevation (Marullo et al., 2013; Wisnovsky et al., 2013).

Platinum-based antineoplastics irreversibly damage the cochlear hair cells starting in the basal turn—the outer hair cells appear to be more susceptible to this class of drug than other cell types in the cochlear duct, including the inner hair cells (Hinojosa et al., 1995; Li et al., 2004; Rybak et al., 2007). However, cisplatin-induced insult could extend beyond the hair cells and damage cells of the *stria vascularis*, a critical organ within the cochlea that is essential for maintaining the endocochlear potential and function of the cochlea (Laurell and Engstrom, 1989; Laurell et al., 2007). Although, damage to mostly outer hair cells is observed when low doses of cisplatin are used in rodents (Laurell and Engstrom, 1989; Cardinaal et al., 2000; Laurell et al., 2000; Park et al., 2002).

Routes of cisplatin entry into the hair cell could include the organic cation transporter Oct2 or the influx copper transporter Ctr1 (Riedemann et al., 2007; Ciarimboli et al., 2010; More et al., 2010; Xu et al., 2012). In addition, it was reported that in the absence of hair cell mechanotransduction (MET) cisplatin-induced hair cell death is reduced in zebrafish neuromast (Thomas et al., 2013; Stawicki et al., 2014). Gentamicin, which is bigger in size and weight than cisplatin, is known to permeate MET channels (Marcotti et al., 2005; Alharazneh et al., 2011; Vu et al., 2013); similarly, it is possible that cisplatin can permeate hair cell MET channels, although other routes could exist (Thomas et al., 2013). Using the zebrafish lateral line system, we test whether cisplatin affects hair cell MET currents, which might implicate its interaction with MET channels.

Attempts to find and develop otoprotective strategies for platinum-based drugs have been ongoing. One area of interest is antioxidant molecules. These include N-acetyl-cysteine (Feghali et al., 2001), alpha-lipoic acid (Kim et al., 2014), D-methionine (Lorito et al., 2011) and sodium thiosulfate (Muldoon et al., 2000). The most important consideration is to find a protection method or a drug that does not compromise the anti-tumor actions of the platinum-based drugs. For that reason, using mdivi-1, an inhibitor of the mitochondrial fission protein Drp1, could be a promising strategy to mitigate cisplatin-induced ototoxicity (Qian et al., 2015). One interesting aspect of mdivi-1 is that it has been reported to increase the apoptosis of tumor cells that are resistant to cisplatin (Qian et al., 2014). In general, mitochondrial dynamics were found to modulate antineoplastic activity of cisplatin (Qian et al., 2015; Han et al., 2017). Interestingly, cisplatin-induced tubular cell apoptosis and acute kidney injury were reduced by mdivi-1 (Brooks et al., 2009). Some recent work has shown promise for mdivi-1 in ameliorating the adverse effects of ototoxic aminoglycosides on hair cells of the inner ear (Nuttall et al., 2015). Here we test whether mdivi-1 could protect hair cells against cisplatin toxicity using the zebrafish lateral line system.

## Materials and Methods

### Animals

Experiments were conducted using the Tübingen strain of zebrafish of either sex provided by the McDermott zebrafish core facility. Transgenic zebrafish stably expressing GFP in the hair cell body (*pvalb3b*::GFP) were previously generated (McDermott et al., 2010), and *cdh23^tj264a^* mutant (Söllner et al., 2004) was a kind gift from Dr. Teresa Nicolson (Oregon Health and Science University). Fish were maintained and bred at 28°C according to standard procedures (Nüsslein-Volhard and Dahm, 2002). This study was carried out in accordance with the recommendations in the Guide for the Care and Use of Laboratory Animals of the National Institutes of Health and animal welfare guidelines of the Committee of Case Western Reserve University (CWRU), USA. The protocol was approved by the Institutional Animal Care and Use Committee at CWRU (Protocol Number: 2012-0187).

### Cisplatin Treatment

Zebrafish larvae at days post fertilization (dpf) 5–6, were placed in varying concentrations of cisplatin (50–200 μM, ThermoFisher Scientific, Waltham, MA, USA) and/or mdivi-1 (1–10 μM, Enzo Life Sciences, Farmingdale, NY, USA) overnight for 16 h. The next day, the larvae were transferred to another dish, anesthetized with MS-222 (Sigma-Aldrich, St. Louis, MO, USA), and secured in a recording chamber using strands of dental floss tie downs (Ricci and Fettiplace, 1997) and placed under the microscope, an upright Olympus BX51WI microscope equipped with 100× 1NA objective for observation. To assess viability, blood flow and heart rate were visually monitored. Images were observed with a Grasshopper3 CMOS camera (Point Grey, Richmond, BC, Canada) and captured with manufacturer provided software. Starting with the eye neuromasts and moving caudal, the number of hair bundles were counted in approximately 10 neuromasts per fish.

### FM1-43 Labeling and Image Analyses

After overnight treatment with cisplatin and/or Mdivi-1, fish were placed into wells containing FM1-43 (ThermoFisher Scientific, Waltham, MA, USA) in fish water. After 30 s, fish were transferred to fish solution containing MS-222 and BSA. The larvae were then secured in a recording chamber and placed under the microscope for imaging as described above. Approximately 3–4 neuromasts were imaged, and maximal projection images were generated using ImageJ (NIH, Bethesda, MD, USA). For lateral line neuromasts, raw images were gathered using an Olympus BX51WI microscope and a Grasshopper3 CMOS camera as described above. Fluorescence measurements were obtained using ImageJ. A region of interest was used to obtain measurements from the cells in each neuromast (I_cell_) and an area without cells (I_background_) in the same image. Fluorescence intensity of FM1-43FX (I_load_) for each neuromast was normalized (I_load_ = I_cell_ − I_background_).

### Recordings of Neuromast Microphonic Potential in Zebrafish

We anesthetized zebrafish larvae (5–7 dpf) using MS-222 dissolved in a standard bath solution containing (in mM): NaCl (120), KCl (2), HEPES (10), CaCl_2_ (2), NaH_2_PO_4_ (0.7), adjusted to pH ~7.2. The larvae were secured in a recording chamber and placed under the microscope for observation as described above. Viability, blood flow and heart rate of larvae were visually monitored. Images were observed with a Grasshopper3 CMOS camera and captured with manufacturer provided software. We recorded from posterior neuromasts; kinocilia tufts were deflected with a fluid jet (Nicolson et al., 1998; Trapani and Nicolson, 2010) delivered via a glass pipette with a diameter of approximately 5–7 μm and controlled by HSPC-1 (ALA Scientific Instruments, Farmingdale, NY, USA). Fluid jet pipette was placed approximately 75 μm near the neuromast and used to deliver sinusoidal stimuli of 50 Hz frequency. The microphonic potentials were recorded at room temperature (22°C). We used borosilicate glass electrodes with a resistance of 3–6 MΩ, which were filled with standard bath solution and placed near the apical edges of the lateral line neuromasts. We recorded microphonic potentials using a PC-505B amplifier (Warner Instruments, Hamden, CT, USA) and a PCI-6221 digitizer (National Instruments, Austin, TX, USA). Microphonic potentials were amplified by 20 (SIM983, Stanford Research, Sunnyvale, CA, USA), measured by a jClamp (Scisoft, Yale University, New Haven, CT, USA) in a current-clamp mode, and low-pass filtered at 100 Hz. All records represent an average of at least 500 trials.

### Statistics

All statistical analyses were performed using GraphPad Prism 7. Data are reported as mean ± SEM. Comparisons between groups were analyzed by ANOVA with Tukey *post hoc* testing.

## Results and Discussion

### Mechanotransduction Potentiates Cisplatin-Induced Hair Cell Death

**Figure 1:**
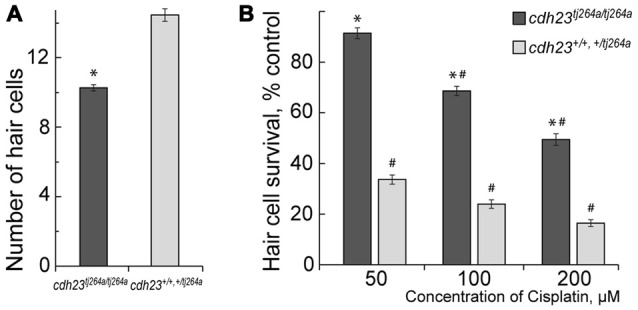
Mechanotransduction (MET) potentiates cisplatin-induced hair cell death. **(A)** Untreated *cdh23^tj264a/tj264a^* have fewer hair cells per neuromast when compared to wild-type and heterozygous fish. **(B)** When treated with increasing concentrations of cisplatin, *cdh23* mutants, which do not have functional MET, have significantly greater hair cell survival in comparison to wild-type or heterozygous animals, which have normal MET. Data are mean, error bars indicate SEM. *p < 0.001, in comparison to wild-type and normal heterozygous larvae within the same treatment concentration. ^#^*p* < 0.001, in comparison to untreated controls within larvae of the same genotype (see Supplementary Table S1).

Our data show that functional MET potentiate cisplatin-induced hair cell toxicity in lateral line neuromasts in a zebrafish (Figure 1), in accordance with published reports (Thomas et al., 2013; Stawicki et al., 2014). *cdh23^tj264a/tj264a^* mutant zebrafish do not have functional MET in hair cells, because Cdh23 is an integral part of mechanosensitive stereocilia bundles in hair cells (Siemens et al., 2004; Söllner et al., 2004; Kazmierczak et al., 2007; Indzhykulian et al., 2013). Notably, *cdh23* mutants have smaller numbers of hair cells per neuromast in comparison to wild-type or heterozygous fish (Figure 1). Despite the fact that treatment with 50 μM of cisplatin did not significantly change the number of hair cells in neuromasts of *cdh23^tj264a/tj264a^* zebrafish, whereas in wild-type fish this dose of cisplatin considerably reduced the number of hair cells (Figure 1A). This result indicates that MET channels may be involved in cisplatin entry into the hair cell. Alternatively, cisplatin entry into the hair cell is largely independent of the MET channel, but the ion flow carried out by functional MET potentiates cisplatin-induced damage.

### Cisplatin and Mechanotransduction in Neuromast Hair Cells

**Figure 2:**
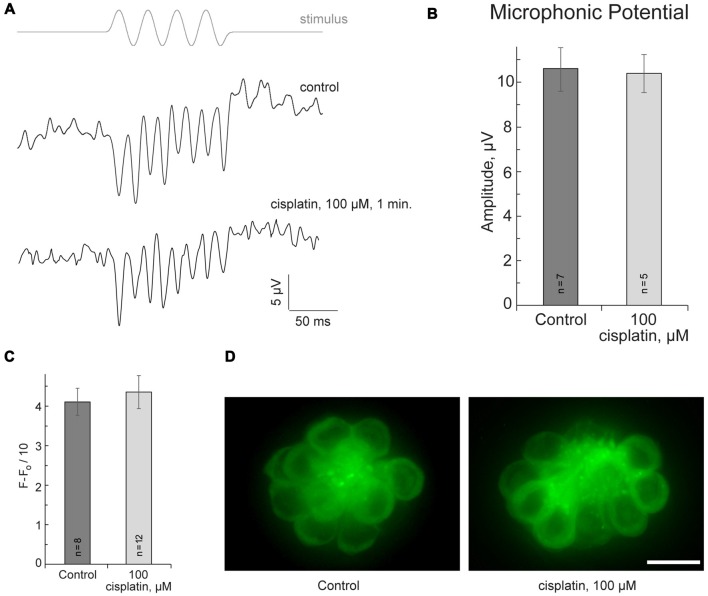
Cisplatin does not affect MET in neuromast hair cells. **(A)** Neuromast microphonic potentials are not affected after 100-μM-cisplatin application. The top trace shows pressure applied to the stimulating puff pipette. **(B)** Summary of microphonic potential peak-to-peak amplitudes at twice the stimulus frequency obtained from lateral line neuromasts (controls and after 100-μM-cisplatin application). **(C)** Summary of fluorescent signal of FM1-43FX in live lateral line neuromasts of control and after 100-μM-cisplatin application. **(D)** Representative maximum-intensity projection images of FM1-43FX-treated live neuromasts: control and after 100-μM-cisplatin. Data are mean, error bars indicate SEM. *n* = 5–12 larvae (noted on bar graphs, from three to six clutches) per data point. Scale bar: 10 μm.

If cisplatin enters hair cells via MET channels, it could interact with the channel directly and attenuate ion flow through the channel. To test this hypothesis, the microphonic potentials of neuromast hair cells (Figures 2A,B) were measured with and without application of 50 μM or 100 μM of cisplatin. The microphonic potential is an evoked electrical potential elicited by hair bundle deflections. The microphonic potential results from modulation of the cationic current flowing into stimulated hair cells via functional MET channels. Our results show that microphonic potentials were not affected by cisplatin application (Figures 2A,B). An alternate approach using FM1-43FX was also employed to test the hypothesis. FM1–43FX is a derivative of FM1-43, an amphipathic styryl dye that is known to rapidly accumulate in sensory hair cells via the MET channels that are partially open at rest in non-stimulated hair bundles (Gale et al., 2001; Meyers et al., 2003). Loading of FM1-43FX in live hair cells of lateral line neuromasts of controls and after 100-μM-cisplatin was not significantly different (Figures 2C,D). Our results did not reveal any evidence that cisplatin enters hair cells via MET channels. It is known that aminoglycosides enter hair cells via MET channels and are permeant blockers of these channels. Our results, however, do not rule out the possibility cisplatin may enter hair cells via the MET channel but this amount may not be sufficient to affect measured microphonic potentials.

When MET is functional, substantial amounts of calcium can enter hair cells through MET channels. Intracellular calcium balance is critical for hair cell function; it was found that calcium homeostasis is rapidly disrupted following ototoxic aminoglycoside exposure (Esterberg et al., 2014). It is possible that hair cell mitochondria continuously buffer calcium entering cell via functional MET channels, causing hair cells to become more vulnerable to toxic insult. Drugs that could reduce mitochondrial stress and/or protect mitochondria in other ways, may potentially increase hair cell viability when faced with ototoxic drugs.

### Mitochondrial Division Inhibitor 1 Protects against Cisplatin-Induced Hair Cell Death

**Figure 3:**
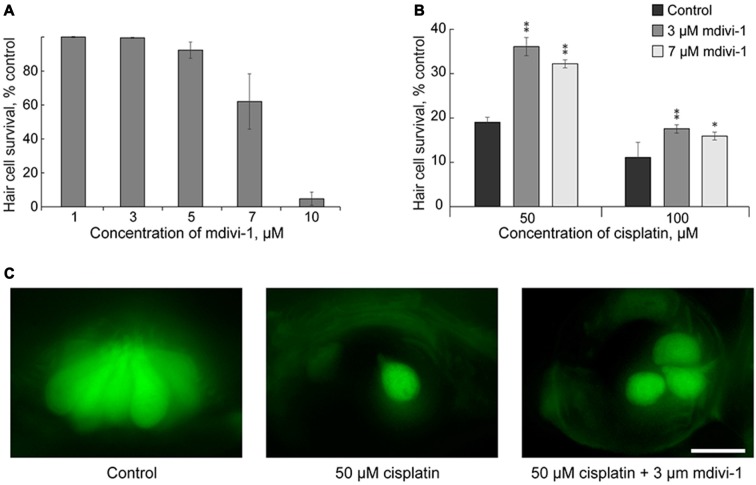
Mdivi-1 protects against cisplatin-induced hair cell death. **(A)** Concentrations of mitochondrial division inhibitor mdivi-1 between 1–5 μM are well tolerated by zebrafish; whereas 10 μM of mdivi-1 is toxic to hair cells (*n* = 5 larvae per data point, from three clutches). **(B)** Application of 3 or 7 μM of mdivi-1 allowed significantly more hair cells to survive treatment with 50 and 100 μM of cisplatin. **(C)** Representative maximum-intensity projection images of *pvalb3b*::GFP neuromast hair cells treated with 50-μM-cisplatin and/or 50-μM-mdivi-1 (middle and right images). Data are mean, error bars indicate SEM. **p < 0.001 and *p < 0.05, in comparison to no mdivi-1 treatment within the same cisplatin concentration (see Supplementary Table S1). Scale bar: 10 μm.

Here we tested whether mdivi-1 can protect hair cells against cisplatin induced toxicity in neuromast hair cells. Mdivi-1 is an inhibitor of mitochondrial division that selectively attenuates dynamin-related protein 1 activity, a fission protein that involved in the constriction and cleavage of mitochondria (Cassidy-Stone et al., 2008). First, we tested different doses of mdivi-1 for neuromast hair cell toxicity. High doses of mdivi-1, more than 10 μM, were toxic to the 5–6 dpf larvae (Figure 3A); therefore, we used lower doses of mdivi-1, 3 and 7 μM. Our data show that these doses of mdivi-1 protected hair cells of lateral line neuromast against toxicity of 50 μM of cisplatin (Figures 3B,C). These data demonstrated that modulating mitochondria dynamics may increase viability of hair cells against cisplatin toxicity in a zebrafish model. This finding is interesting also because it is known that mdivi-1 assists the abilities of cisplatin to trigger apoptosis in certain platinum-resistant tumor cells (Qian et al., 2014). Future studies, incorporating mammalian models, will be of further value in corroborating our results and revealing the mechanism of mdivi-1-mediated protection.

## Conclusion

MET potentiates cisplatin-induced damage of neuromast hair cells. However, cisplatin, in contrast to aminoglycosides, does not affect MET of neuromast hair cells. Our data suggests that mitochondrial protection may prevent cisplatin-induced damage to hair cells.

## Author Contributions

JWV and RS: conceived and designed the experiments and wrote the article. JWV, RS and SNW: performed the experiments and analyzed the data. JWV, SNW, SRG, ARD, BMM, KNA and RS: discussion and contributed reagents, materials, animal work.

## Conflict of Interest Statement

The authors declare that the research was conducted in the absence of any commercial or financial relationships that could be construed as a potential conflict of interest.

## References

[B1] Alharazneh A. Luk L. Huth M. Monfared A. Steyger P. S. Cheng A. G.. (2011). Functional hair cell mechanotransducer channels are required for aminoglycoside ototoxicity. PLoS One 6:e22347. 10.1371/journal.pone.0022347 21818312 PMC3144223

[B2] Brock P. R. Knight K. R. Freyer D. R. Campbell K. C. Steyger P. S. Blakley B. W.. (2012). Platinum-induced ototoxicity in children: a consensus review on mechanisms, predisposition, and protection, including a new International Society of Pediatric Oncology Boston ototoxicity scale. J. Clin. Oncol. 30, 2408–2417. 10.1200/JCO.2011.39.1110 22547603 PMC3675696

[B3] Brooks C. Wei Q. Cho S. G. Dong Z. (2009). Regulation of mitochondrial dynamics in acute kidney injury in cell culture and rodent models. J. Clin. Invest. 119, 1275–1285. 10.1172/JCI37829 19349686 PMC2673870

[B4] Cardinaal R. M. de Groot J. C. Huizing E. H. Veldman J. E. Smoorenburg G. F. (2000). Histological effects of co-administration of an ACTH_(4–9)_ analogue, ORG 2766, on cisplatin ototoxicity in the albino guinea pig. Hear. Res. 144, 157–167. 10.1016/s0378-5955(00)00061-7 10831874

[B5] Cassidy-Stone A. Chipuk J. E. Ingerman E. Song C. Yoo C. Kuwana T.. (2008). Chemical inhibition of the mitochondrial division dynamin reveals its role in Bax/Bak-dependent mitochondrial outer membrane permeabilization. Dev. Cell 14, 193–204. 10.1016/j.devcel.2007.11.019 18267088 PMC2267902

[B6] Ciarimboli G. Deuster D. Knief A. Sperling M. Holtkamp M. Edemir B.. (2010). Organic cation transporter 2 mediates cisplatin-induced oto- and nephrotoxicity and is a target for protective interventions. Am. J. Pathol. 176, 1169–1180. 10.2353/ajpath.2010.090610 20110413 PMC2832140

[B7] Cornelison T. L. Reed E. (1993). Nephrotoxicity and hydration management for cisplatin, carboplatin and ormaplatin. Gynecol. Oncol. 50, 147–158. 10.1006/gyno.1993.1184 8375728

[B8] Devarajan P. Savoca M. Castaneda M. P. Park M. S. Esteban-Cruciani N. Kalinec G.. (2002). Cisplatin-induced apoptosis in auditory cells: role of death receptor and mitochondrial pathways. Hear. Res. 174, 45–54. 10.1016/s0378-5955(02)00634-2 12433395

[B9] Esterberg R. Hailey D. W. Rubel E. W. Raible D. W. (2014). ER-mitochondrial calcium flow underlies vulnerability of mechanosensory hair cells to damage. J. Neurosci. 34, 9703–9719. 10.1523/JNEUROSCI.0281-14.2014 25031409 PMC4099547

[B11] Feghali J. G. Liu W. Van De Water T. R. (2001). L-N-acetyl-cysteine protection against cisplatin-induced auditory neuronal and hair cell toxicity. Laryngoscope 111, 1147–1155. 10.1097/00005537-200107000-00005 11568534

[B12] Francis S. P. Cunningham L. L. (2017). Non-autonomous cellular responses to ototoxic drug-induced stress and death. Front. Cell. Neurosci. 11:252. 10.3389/fncel.2017.00252 28878625 PMC5572385

[B13] Gale J. E. Marcotti W. Kennedy H. J. Kros C. J. Richardson G. P. (2001). FM1–43 dye behaves as a permeant blocker of the hair-cell mechanotransducer channel. J. Neurosci. 21, 7013–7025. 11549711 10.1523/JNEUROSCI.21-18-07013.2001 PMC6762973

[B14] Han X. J. Shi S. L. Wei Y. F. Jiang L. P. Guo M. Y. Wu H. L.. (2017). Involvement of mitochondrial dynamics in the antineoplastic activity of cisplatin in murine leukemia L1210 cells. Oncol. Rep. 38, 985–992. 10.3892/or.2017.5765 28677814

[B15] Hinojosa R. Riggs L. C. Strauss M. Matz G. J. (1995). Temporal bone histopathology of cisplatin ototoxicity. Am. J. Otol. 16, 731–740. 8572135

[B16] Huang H. Zhu L. Reid B. R. Drobny G. P. Hopkins P. B. (1995). Solution structure of a cisplatin-induced DNA interstrand cross-link. Science 270, 1842–1845. 10.1126/science.270.5243.1842 8525382

[B17] Indzhykulian A. A. Stepanyan R. Nelina A. Spinelli K. J. Ahmed Z. M. Belyantseva I. A.. (2013). Molecular remodeling of tip links underlies mechanosensory regeneration in auditory hair cells. PLoS Biol. 11:e1001583. 10.1371/journal.pbio.1001583 23776407 PMC3679001

[B18] Jamieson E. R. Lippard S. J. (1999). Structure, recognition, and processing of cisplatin-DNA adducts. Chem. Rev. 99, 2467–2498. 11749487 10.1021/cr980421n

[B19] Karasawa T. Steyger P. S. (2015). An integrated view of cisplatin-induced nephrotoxicity and ototoxicity. Toxicol. Lett. 237, 219–227. 10.1016/j.toxlet.2015.06.012 26101797 PMC4516600

[B20] Kazmierczak P. Sakaguchi H. Tokita J. Wilson-Kubalek E. M. Milligan R. A. Muller U.. (2007). Cadherin 23 and protocadherin 15 interact to form tip-link filaments in sensory hair cells. Nature 449, 87–91. 10.1038/nature06091 17805295

[B21] Kim J. Cho H.-J. Sagong B. Kim S.-J. Lee J.-T. So H.-S.. (2014). α-lipoic acid protects against cisplatin-induced ototoxicity via the regulation of MAPKs and proinflammatory cytokines. Biochem. Biophys. Res. Commun. 449, 183–189. 10.1016/j.bbrc.2014.04.118 24796665

[B22] Kopke R. D. Liu W. Gabaizadeh R. Jacono A. Feghali J. Spray D.. (1997). Use of organotypic cultures of Corti’s organ to study the protective effects of antioxidant molecules on cisplatin-induced damage of auditory hair cells. Am. J. Otol. 18, 559–571. 9303151

[B23] Laurell G. Ekborn A. Viberg A. Canlon B. (2007). Effects of a single high dose of cisplatin on the melanocytes of the stria vascularis in the guinea pig. Audiol. Neurootol. 12, 170–178. 10.1159/000099020 17259704

[B24] Laurell G. Engstrom B. (1989). The combined effect of cisplatin and furosemide on hearing function in guinea pigs. Hear. Res. 38, 19–26. 10.1016/0378-5955(89)90124-x 2708156

[B25] Laurell G. Viberg A. Teixeira M. Sterkers O. Ferrary E. (2000). Blood-perilymph barrier and ototoxicity: an *in vivo* study in the rat. Acta Otolaryngol. 120, 796–803. 10.1080/000164800750061624 11132710

[B26] Li Y. Womer R. B. Silber J. H. (2004). Predicting cisplatin ototoxicity in children: the influence of age and the cumulative dose. Eur. J. Cancer 40, 2445–2451. 10.1016/j.ejca.2003.08.009 15519518

[B27] Lorito G. Hatzopoulos S. Laurell G. Campbell K. C. M. Petruccelli J. Giordano P.. (2011). Dose-dependent protection on cisplatin-induced ototoxicity—an electrophysiological study on the effect of three antioxidants in the Sprague-Dawley rat animal model. Med. Sci. Monit. 17, BR179–BR186. 10.12659/msm.881894 21804453 PMC3539615

[B28] Marcotti W. van Netten S. M. Kros C. J. (2005). The aminoglycoside antibiotic dihydrostreptomycin rapidly enters mouse outer hair cells through the mechano-electrical transducer channels. J. Physiol. 567, 505–521. 10.1113/jphysiol.2005.085951 15994187 PMC1474200

[B29] Marullo R. Werner E. Degtyareva N. Moore B. Altavilla G. Ramalingam S. S.. (2013). Cisplatin induces a mitochondrial-ROS response that contributes to cytotoxicity depending on mitochondrial redox status and bioenergetic functions. PLoS One 8:e81162. 10.1371/journal.pone.0081162 24260552 PMC3834214

[B30] McDermott B. M. Jr. Asai Y. Baucom J. M. Jani S. D. Castellanos Y. Gomez G.. (2010). Transgenic labeling of hair cells in the zebrafish acousticolateralis system. Gene Expr. Patterns 10, 113–118. 10.1016/j.gep.2010.01.001 20085825 PMC2863287

[B31] Meyers J. R. MacDonald R. B. Duggan A. Lenzi D. Standaert D. G. Corwin J. T.. (2003). Lighting up the senses: FM1–43 loading of sensory cells through nonselective ion channels. J. Neurosci. 23, 4054–4065. 12764092 10.1523/JNEUROSCI.23-10-04054.2003 PMC6741082

[B32] More S. S. Akil O. Ianculescu A. G. Geier E. G. Lustig L. R. Giacomini K. M. (2010). Role of the copper transporter, CTR1, in platinum-induced ototoxicity. J. Neurosci. 30, 9500–9509. 10.1523/JNEUROSCI.1544-10.2010 20631178 PMC2949060

[B33] Muldoon L. L. Pagel M. A. Kroll R. A. Brummett R. E. Doolittle N. D. Zuhowski E. G.. (2000). Delayed administration of sodium thiosulfate in animal models reduces platinum ototoxicity without reduction of antitumor activity. Clin. Cancer Res. 6, 309–315. 10656463

[B34] Nicolson T. Rüsch A. Friedrich R. W. Granato M. Ruppersberg J. P. Nüsslein-Volhard C. (1998). Genetic analysis of vertebrate sensory hair cell mechanosensation: the zebrafish circler mutants. Neuron 20, 271–283. 10.1016/s0896-6273(00)80455-9 9491988

[B35] Nüsslein-Volhard C. Dahm R. (2002). Zebrafish: A Practical Approach. New York, NY: Oxford University Press.

[B36] Nuttall A. Foster S. Zhang Y. Wilson T. (2015). “Points, strands and donuts: the mitochondria dynamics of cochlear cells,” in Association for Research in Otolaryngology 38th MedWinter Meeting (Barltimore, MD).

[B37] Park M. S. De Leon M. Devarajan P. (2002). Cisplatin induces apoptosis in LLC-PK1 cells via activation of mitochondrial pathways. J. Am. Soc. Nephrol. 13, 858–865. 11912244 10.1681/ASN.V134858

[B38] Qian W. Salamoun J. Wang J. Roginskaya V. Van Houten B. Wipf P. (2015). The combination of thioxodihydroquinazolinones and platinum drugs reverses platinum resistance in tumor cells by inducing mitochondrial apoptosis independent of Bax and Bak. Bioorg. Med. Chem. Lett. 25, 856–863. 10.1016/j.bmcl.2014.12.072 25582599 PMC4318771

[B39] Qian W. Wang J. Roginskaya V. McDermott L. A. Edwards R. P. Stolz D. B.. (2014). Novel combination of mitochondrial division inhibitor 1 (mdivi-1) and platinum agents produces synergistic pro-apoptotic effect in drug resistant tumor cells. Oncotarget 5, 4180–4194. 10.18632/oncotarget.1944 24952704 PMC4147315

[B40] Ricci A. J. Fettiplace R. (1997). The effects of calcium buffering and cyclic AMP on mechano-electrical transduction in turtle auditory hair cells. J. Physiol. 501, 111–124. 10.1111/j.1469-7793.1997.111bo.x 9174998 PMC1159508

[B41] Riedemann L. Lanvers C. Deuster D. Peters U. Boos J. Jürgens H.. (2007). Megalin genetic polymorphisms and individual sensitivity to the ototoxic effect of cisplatin. Pharmacogenomics J. 8, 23–28. 10.1038/sj.tpj.6500455 17457342

[B42] Rybak L. P. Mukherjea D. Jajoo S. Ramkumar V. (2009). Cisplatin ototoxicity and protection: clinical and experimental studies. Tohoku J. Exp. Med. 219, 177–186. 10.1620/tjem.219.177 19851045 PMC2927105

[B44] Rybak L. P. Whitworth C. A. Mukherjea D. Ramkumar V. (2007). Mechanisms of cisplatin-induced ototoxicity and prevention. Hear. Res. 226, 157–167. 10.1016/j.heares.2006.09.015 17113254

[B43] Rybak L. P. Whitworth C. Somani S. (1999). Application of antioxidants and other agents to prevent cisplatin ototoxicity. Laryngoscope 109, 1740–1744. 10.1097/00005537-199911000-00003 10569399

[B45] Schacht J. Talaska A. E. Rybak L. P. (2012). Cisplatin and aminoglycoside antibiotics: hearing loss and its prevention. Anat. Rec. 295, 1837–1850. 10.1002/ar.22578 23045231 PMC3596108

[B46] Siemens J. Lillo C. Dumont R. A. Reynolds A. Williams D. S. Gillespie P. G.. (2004). Cadherin 23 is a component of the tip link in hair-cell stereocilia. Nature 428, 950–955. 10.1038/nature02483 15057245

[B47] Söllner C. Rauch G. J. Siemens J. Geisler R. Schuster S. C. Müller U.. (2004). Mutations in cadherin 23 affect tip links in zebrafish sensory hair cells. Nature 428, 955–959. 10.1038/nature02484 15057246

[B48] Stawicki T. M. Owens K. N. Linbo T. Reinhart K. E. Rubel E. W. Raible D. W. (2014). The zebrafish merovingian mutant reveals a role for pH regulation in hair cell toxicity and function. Dis. Model. Mech. 7, 847–856. 10.1242/dmm.016576 24973752 PMC4073274

[B49] Thomas A. J. Hailey D. W. Stawicki T. M. Wu P. Coffin A. B. Rubel E. W.. (2013). Functional mechanotransduction is required for cisplatin-induced hair cell death in the zebrafish lateral line. J. Neurosci. 33, 4405–4414. 10.1523/JNEUROSCI.3940-12.2013 23467357 PMC3666553

[B50] Trapani J. G. Nicolson T. (2010). Physiological recordings from zebrafish lateral-line hair cells and afferent neurons. Methods Cell Biol. 100, 219–231. 10.1016/B978-0-12-384892-5.00008-6 21111219

[B51] Vu A. A. Nadaraja G. S. Huth M. E. Luk L. Kim J. Chai R.. (2013). Integrity and regeneration of mechanotransduction machinery regulate aminoglycoside entry and sensory cell death. PLoS One 8:e54794. 10.1371/journal.pone.0054794 23359017 PMC3554584

[B52] Wisnovsky S. P. Wilson J. J. Radford R. J. Pereira M. P. Chan M. R. Laposa R. R.. (2013). Targeting mitochondrial DNA with a platinum-based anticancer agent. Chem. Biol. 20, 1323–1328. 10.1016/j.chembiol.2013.08.010 24183971 PMC4082333

[B53] Wong E. Giandomenico C. M. (1999). Current status of platinum-based antitumor drugs. Chem. Rev. 99, 2451–2466. 10.1021/cr980420v 11749486

[B54] Xu X. Ren H. Zhou B. Zhao Y. Yuan R. Ma R.. (2012). Prediction of copper transport protein 1 (CTR1) genotype on severe cisplatin induced toxicity in non-small cell lung cancer (NSCLC) patients. Lung Cancer 77, 438–442. 10.1016/j.lungcan.2012.03.023 22516052

