# Supplementary material for: Inhibition of Mitochondrial Division Attenuates Cisplatin-Induced Toxicity in the Neuromast Hair Cells
Source: Front Cell Neurosci. 2017 Dec 12;11:393. doi: 10.3389/fncel.2017.00393 (PMC5732985; doi:10.3389/fncel.2017.00393)
Supplement: Supplementary file 1 [file Table_1.docx]

Table 1S **A** and **B**: Adjusted P values, Tukey’s multiple comparisons tests, used in (**A**) Fig. 1 and (**B**) Fig.3. The number of independent experiments underlying each data point: (**A**) *n* = 5 for *cdh23^+/+, +/ tj264a^* groups; *n* = 4 for *cdh23^tj264a/ tj264a^* groups; and, (**B**) *n* = 8. For each experiment, 1 – 4 larvae were used per data point. Total number of larvae used per data point (or group) noted next to each group. For each larvae average number of hair cells in 7 - 12 neuromasts were used.

**A**

| *P Values* | *Comparisons* | |
| --- | --- | --- |
|  | *Group (total number of larvae)* | *Group / (total number of larvae)* |
| 4.24E-05 | *cdh23^tj264a/ tj264a^* (12) | *cdh23^+/+, +/ tj264a^* (9) |
| 1.11E-05 | *cdh23^tj264a/ tj264a^*, 50 µM cisplatin (15) | *cdh23^+/+, +/ tj264a^*, 50 µM cisplatin (10) |
| 2.25E-04 | *cdh23^tj264a/ tj264a^*, 100 µM cisplatin (18) | *cdh23^+/+, +/ tj264a^*, 100 µM cisplatin (10) |
| 1.52E-09 | *cdh23^tj264a/ tj264a^*, 200 µM cisplatin (17) | *cdh23^+/+, +/ tj264a^*, 200 µM cisplatin (10) |
|  |  |  |
| 8.31E-01 | *cdh23^tj264a/ tj264a^* (12) | *cdh23^tj264a/ tj264a^*, 50 µM cisplatin (18) |
| < 1E-15 | *cdh23^+/+, +/ tj264a^* (9) | *cdh23^+/+, +/ tj264a^*, 50 µM cisplatin (10) |
| 4.12E-05 | *cdh23^tj264a/ tj264a^* (12) | *cdh23^tj264a/ tj264a^*, 100 µM cisplatin (18) |
| < 1E-15 | *cdh23^+/+, +/ tj264a^* (9) | *cdh23^+/+, +/ tj264a^*, 100 µM cisplatin (10) |
| 3.19E-09 | *cdh23^tj264a/ tj264a^* (12) | *cdh23^tj264a/ tj264a^*, 200 µM cisplatin (17) |
| < 1E-15 | *cdh23^+/+, +/ tj264a^* (9) | *cdh23^+/+, +/ tj264a^*, 200 µM cisplatin (10) |

**B**

| *P Values* | *Comparisons* | |
| --- | --- | --- |
|  | *Group (total number of larvae)* | *Group (total number of larvae)* |
| 1.62E-04 | 50 µM cisplatin (22) | 50 µM cisplatin + 3 µM mdivi-1 (20) |
| 9.79E-04 | 50 µM cisplatin (22) | 50 µM cisplatin + 7 µM mdivi-1 (11) |
| 1.11E-04 | 100 µM cisplatin (18) | 100 µM cisplatin + 3 µM mdivi-1 (18) |
| 1.40E-02 | 100 µM cisplatin (18) | 100 µM cisplatin + 7 µM mdivi-1 (10) |
